# A tunable anthranilate-inducible gene expression system for *Pseudomonas* species

**DOI:** 10.1007/s00253-020-11034-8

**Published:** 2020-12-03

**Authors:** Lena Hoffmann, Michael-Frederick Sugue, Thomas Brüser

**Affiliations:** Institute of Microbiology, Leibniz University Hannover, Herrenhäuser Str. 2, 30419 Hannover, Germany

**Keywords:** Anthranilate, Recombinant expression, Gene regulation, *Pseudomonas fluorescens*, *Pseudomonas aeruginosa*

## Abstract

**Abstract:**

Pseudomonads are among the most common bacteria in soils, limnic ecosystems, and human, animal, or plant host environments, including intensively studied species such as *Pseudomonas aeruginosa*, *P. putida*, or *P. fluorescens*. Various gene expression systems are established for some species, but there is still a need for a simple system that is suitable for a wide range of pseudomonads and that can be used for physiological applications, i.e., with a tuning capacity at lower expression levels. Here, we report the establishment of the anthranilate-dependent *P*_*antA*_ promoter for tunable gene expression in pseudomonads. During studies on *P. fluorescens*, we constructed an anthranilate-inducible AntR/*P*_*antA*_-based expression system, named pUCP20-ANT, and used GFP as reporter to analyze gene expression. This system was compared with the rhamnose-inducible RhaSR/*P*_*rhaB*_-based expression system in an otherwise identical vector background. While the rhamnose-inducible system did not respond to lower inducer concentrations and always reached high levels over time when induced, expression levels of the pUCP20-ANT system could be adjusted to a range of distinct lower or higher levels by variation of anthranilate concentrations in the medium. Importantly, the anthranilate-inducible expression system worked also in strains of *P. aeruginosa* and *P. putida* and therefore will be most likely useful for physiological and biotechnological purposes in a wide range of pseudomonads.

**Key points:**

*• We established an anthranilate-inducible gene expression system for pseudomonads.*

*• This system permits tuning of gene expression in a wide range of pseudomonads.*

*• It will be very useful for physiological and biotechnological applications.*

**Supplementary Information:**

The online version contains supplementary material available at 10.1007/s00253-020-11034-8.

## Introduction

Pseudomonads play important roles in many ecosystems, and they often occur in mutualistic or pathogenic life styles associated with plants, animals, or humans (Peix et al. 2018). Prominent model organisms are, among others, the plant growth-promoting *P. fluorescens*, the human opportunistic pathogen *P. aeruginosa*, and *P. putida*, which is used for bioremediation and biotechnology (Diggle and Whiteley 2020; Kim and Anderson 2018; Poblete-Castro et al. 2012). Pseudomonads are frequently isolated and often there is the wish to produce functional proteins in these strains to study complementation, interaction, localization, transport, or other physiological aspects related to these proteins. For that purpose, there is a need for a regulated, tightly controlled expression system that is simple and tunable in diverse pseudomonads. Albeit a number of expression systems have been reported for pseudomonads, no single system meets these criteria so far. Benzoate/toluate-inducible (Mermod et al. 1986) as well as a dicyclopropylketone-inducible (Smits et al. 2001) systems have been designed to achieve high expression levels (5–10% of the cell protein), but these levels are not useful for physiological studies. For the toluate-inducible system, induction has been demonstrated to switch from uninduced to fully induced at about 1 μM inducer in *P. putida*, and that the promoter has a leakage of about 5% in *P. putida* and *P. aeruginosa* (Mermod et al. 1986). A LacI^q^/*P*_*tac*_-based expression system has been established that is useful for overproduction of proteins, but this system is very leaky under non-induced conditions (de Lorenzo et al. 1993). A broad host range *P*_*lacZ*_ system has been reported, but this promoter gave highest uninduced leaking with pseudomonads (*P. fluorescens*; Khan et al. 2008). Meisner and Goldberg (2016) compared LacI^q^/*P*_*tac*_, AraC/*P*_*araB*_, and RhaSR/*P*_*rhaB*_ promoter systems in *P. aeruginosa* and found that only the RhaSR/*P*_*rhaB*_ system showed tight control of expression, and Jeske and Altenbuchner (2010) had demonstrated earlier with the same expression system that it functions in *P. putida*. However, in no case of these *Pseudomonas* expression systems, the induction was monitored on the cellular level to address the aspect whether or not induction is uniform or whether lower inducer concentrations result in increasing populations of uninduced cells versus fully induced cells. Such “all-or-nothing” systems have been described in *E. coli* for the LacI/*P*_*lacZ*_ and the AraC/*P*_*araB*_ systems (Novick and Weiner 1957; Siegele and Hu 1997). The effect is caused by the upregulation of the uptake transporters in response to the inducer, and the inducer-independent production of the respective transporter gene is required to avoid this phenomenon (Széliová et al. 2016).

We now constructed an expression vector, based on the AntR repressor/*antA* promoter from *P. fluorescens* and the widely used pUCP20 (West et al. 1994), which uses the natural, non-toxic, and cheap anthranilate as inducer, and compared this system with the currently preferred rhamnose-inducible system. Anthranilate is a common intermediate in the bacterial degradation of many aromatic compounds, including the standard amino acid tryptophan (Arora 2015). We demonstrate that anthranilate permits a tunable expression in all cells within the culture, reaching from low to very high expression levels. The anthranilate-based system has several advantages and functions not only in *P. fluorescens* but also in other pseudomonads, such as *P. putida* and *P. aeruginosa*.

## Materials and methods

### Strains and growth conditions

*P. fluorescens* A506, *P. aeruginosa* PAO1, and *P. putida* DSM291^T^, transformed with indicated expression vectors, were used for analyses. *Escherichia coli* DH5α λ *pir*^+^ was used for cloning. *E. coli* was grown on LB medium (1% tryptone, 1% NaCl, 0.5 g/l yeast extract). Pseudomonads were grown either on M9 mineral salt medium (Miller 1972), supplemented with 0.4% glucose as carbon source and 100 μM FeCl_3_ to avoid iron limitation (this medium is referred to as M9 medium throughout this study), or on complex media. King’s B medium was used for *P. putida* (2% peptone, 0.15% K_2_HPO_4_, 0.15% MgSO_4_ × 7H_2_O, 1% glycerol), and LB medium was used for *P. fluorescens* or *P. aeruginosa*. In addition to their regular constituents, all media for pseudomonads were supplemented with 50 mM KH_2_PO_4_/K_2_HPO_4_ (pH 7.0). The *P. fluorescens* and *P. putida* strains were cultivated at 30 °C, and *P. aeruginosa* and *E. coli* strains at 37 °C. For selection, appropriate antibiotics were used in the following final concentrations: 100 μg/ml ampicillin, 50 μg/ml kanamycin, 200 μg/ml carbenicillin.

For promoter studies, 20 ml cultures (100-ml Erlenmeyer flasks with four bottom baffles) were grown for about 16 h in complex media or up to 30 h (M9 medium). Of these precultures, 2 ml was taken and washed three times with 2 ml of the respective medium by centrifugation (16,000×*g* for 2 min at room temperature). The optical density at 600 nm (OD600) of the washed cultures was measured and fresh 100-ml Erlenmeyer flasks with four bottom baffles and 20 ml medium were inoculated to a final OD600 of 0.1. Growth of the cultures was continued to an OD600 of 0.3, and gene expression was induced by the addition of anthranilic acid (1 M stock solution in DMSO) or rhamnose (1 M stock solution in distilled water) to different final concentrations (10 mM, 1 mM, 0.1 mM, 0.01 mM, 0.001 mM, and 0 mM). For repression of the rhamnose system, 1% glucose was added instead of rhamnose. Growth was continued for 1, 2, or 3 more hours.

Growth curves were based on measurements of the OD600 and fluorescence (excitation at 488 nm, emission at 509 nm) in 15-min intervals with cultures that were grown at 30 °C in shaking 96-well plates (culture volume: 100 μl), using the Synergy Mx Multi-Mode Microplate Reader (BioTek Instruments, Winooski, USA).

### Construction of plasmids

A survey of the used and constructed plasmids is given in Table 1. The gene 4486 of *P. fluorescens* A506 (*antR*, see “Results”) was PCR-amplified with the promoter region of *antA* from the genome of *P. fluorescens* A506, using the primer pair Pant-fus-F1 (5′-CAC TCA TTA GGC ACC CCA GGC TGA GAT CCT CCA GGC ACC CCA TAC-3′) and PantA-SpeI-SbfI-R (5′-CGC TCC TGC AGG CAG GTC ACA CTA GTT TGA TCA TGG CTA AAC GGT GAG CC-3′), and fused by fusion PCR with a fragment of pUCP20 (West et al. 1994) that was generated with the primers pUCP20-SapI-f (5′-AGG AAG CGG AAG AGC GCC CAA TAC-3′) and Pant-fus-R1 (5′-GTA TGG GGT GCC TGG AGG ATC TCA GCC TGG GGT GCC TAA TGA GTG-3′) to generate a fragment that then was cloned into pUCP20 using the SapI and HindIII restriction sites. A neo/kan^R^ transcriptional terminator of pK18 (Pridmore 1987) was amplified using primers Ter-SbfI-F (5′-GCT TCC TGC AGG GTT TTC GTT CCA CTG AGC GTC AGA C-3′) and Ter-HindIII-R (5′-GCT CAA GCT TCA GAT TAC GCG CAG AAA AAA AGG-3′), and this PCR-fragment was cloned into the SbfI/HindIII sites, resulting in pUCP20-ANT1.

**Table 1:** Plasmids used in this study

Name	Characteristics	Source
pUCP20	*Escherichia-Pseudomonas* shuttle vector, amp^R^, *lacZ alpha*, pMB1 (ColE1) ori (for *E. coli*), contains pRO1614 region required for replication of pMB1ori in pseudomonads.	West et al. 1994
pK18	pMB1 (ColEI) ori, RP4mob, oriV, oriT, kan^R^, *sacB*	Pridmore 1987
pK18*mobsacB*	Derivative of pK18 with *mob* region of RP4 and a modified *sacB* of *Bacillus subtilis*	Schäfer et al. 1994
pHT01-*gfp*	Expression vector for *B. subtilis*, amp^R^, cam^R^, ColE1 ori, *repA*, *lacI*, *gfp*	Mulvenna et al. 2019
pUCP20-ANT1	pUCP20-based *Escherichia-Pseudomonas* shuttle vector for anthranilate-regulated gene expression, amp^R^, *lacZ alpha*, ColE1 ori (*E. coli*), *antR*, *antA* promoter	This study
pUCP20-ANT1-*gfp*	pUCP20-ANT1 with *gfp* as reporter gene for the anthranilate-regulated *antA* promoter	This study
pUCP20-ANT1-MCS	pUCP20-ANT1 with multiple cloning site of pUCP20	This study
pUCP20-ANT2	As pUCP20-ANT1, but amp^R^ exchanged by kan^R^	This study
pUCP20-ANT2-*gfp*	pUCP20-ANT2 with *gfp* as reporter gene for the anthranilate-regulated *antA* promoter	This study
pUCP20-ANT2-MCS	pUCP20-ANT2 with MCS from pUCP20	This study
pUCP20-RHA1	pUCP20-based *Escherichia-Pseudomonas* shuttle vector for rhamnose-regulated gene expression, amp^R^, *lacZ alpha*, ColE1 ori (*E. coli*), *rhaSR*, *rhaB* promoter	This study
pUCP20-RHA2	As pUCP20-RHA1, but amp^R^ exchanged by kan^R^	This study
pUCP20-RHA2-*gfp*	pUCP20-RHA2 with *gfp* as reporter gene for the rhamnose-regulated *rhaB* promoter	This study

**Fig. 1:**
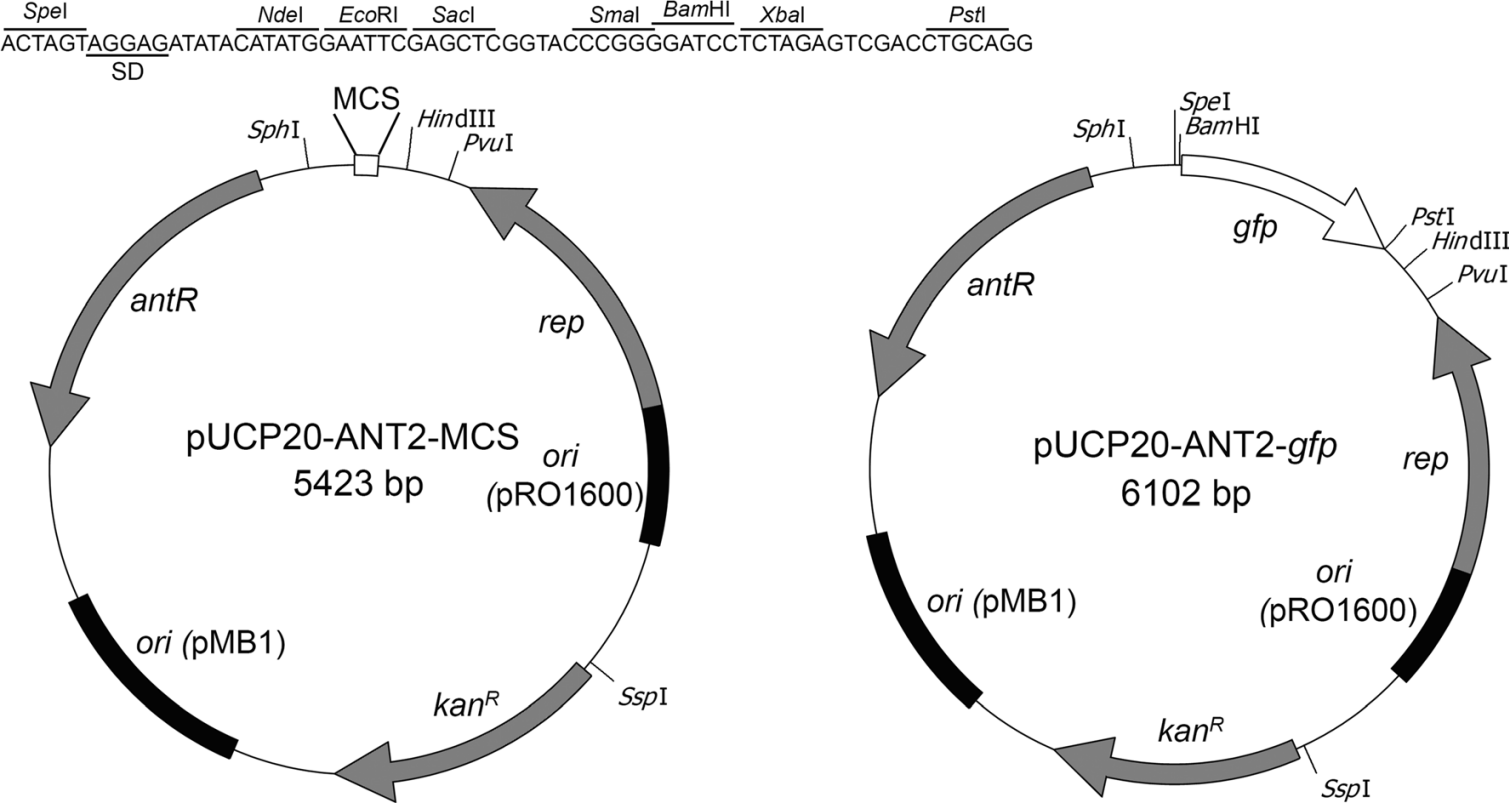
Vector maps of two pUCP20-ANT vectors. Shown are pUCP20-ANT2-MCS and pUCP20-ANT2-*gfp*, which are used for *P. fluorescens* and *P. putida*. For *P. aeruginosa*, the vectors pUCP20-ANT1-MCS and pUCP20-ANT1-*gfp* are used, which differ only in their resistance cassette (amp^R^ instead of kan^R^). The most relevant restriction sites of the MCS are indicated. SD, Shine-Dalgarno site (underlined)

For use in *P. fluorescens*, the resistance cassette was changed from ampicillin to kanamycin. The kan^R^ resistance cassette from pK18mobSacB (Schäfer et al. 1994) was amplified using primers KanR-SspI-SD-F (5′-GCA TAA TAT TAC AGG ATG AGG ATC GTT TCG C-3′) and KanR-BsaI-R (5′-GCT AAC CGC GAG ACC TCA GAA GAA CTC GTC AAG AAG-3′), and the amp^R^ resistance cassette of pUCP20-ANT1 was substituted by this kan^R^ resistance cassette using SspI/BsaI for cloning, resulting in pUCP20-ANT2. For promoter reporter studies, *gfp* was PCR-amplified from pHT01-*gfp* (Mulvenna et al. 2019) using SD-GFP-SpeI-F (5′-GCA GAC TAG TAA AGG AGG AAG GAT CCA TGA G-3′) and GFP-SbfI-R (5′-GCA GCC TGC AGG TTA TTT GTA TAG TTC ATC CAT GCC-3′); the fragment was digested with SpeI/SbfI, and cloned into the corresponding sites of pUCP20-ANT2 to generate pUCP20-ANT2-*gfp*. The multiple cloning site was amplified from pUCP20 using primers MCS-F (5′-GCC GAC TAG TAG GAG ATA TAC ATA TGG AAT TCG AGC TCG GTA CCC GGG GAT CCT C-3′) and MCS-R (5′-TGC CAA GCT TGC ATG CCT GC-3′); the PCR-fragment was digested with SpeI/SbfI, and cloned into the corresponding sites of pUCP20-ANT2 to obtain pUCP20-ANT2-MCS. To use the system for experiments with *P. aeruginosa*, the kanamycin resistance cassette was exchanged by an ampicillin/carbenicillin resistance cassette that was cloned from pUCP20 into pUCP20-ANT2-MCS and pUCP20-ANT2-*gfp* using the SspI/PciI restriction sites, resulting in pUCP20-ANT1-MCS and pUCP20-ANT1-*gfp.* pUCP20-ANT2-MCS and pUCP20-ANT2-*gfp* are shown in Fig. 1 as examples for the herein newly described pUCP20-ANT vectors.

The region including the *rhaSR* operon and the *P*_*rhaB*_ promoter of *E. coli* was amplified from the genome of *E. coli* DH5α λ *pir*^+^ using the primers Prha-fus-F (5′-CAC TCA TTA GGC ACC CCA GGC AAA GAG TGG AAC AAT GCA GG-3′) and Prha-SpeI-SbfI-R (5′-CGC TCC TGC AGG CAG GTC ACA CTA GTT GAA TTT CAT TAC GAC CAG TC-3′), and fused to a PCR-fragment of part of vector backbone that was generated with primers pUCP20-SapI-F (above) and Prha-Fus-R (5′-CCT GCA TTG TTC CAC TCT TTG CCT GGG GTG CCT AAT GAG TG-3′) by fusion PCR. The resulting PCR product was digested by SapI and HindIII and cloned into the corresponding sites of pUCP20 and the terminator region was amplified and integrated as described for the *P*_*antA*_ constructs, resulting in pUCP20-RHA1.

To exchange the resistance cassette by kan^R^, the abovementioned SspI/BsaI-digested kan^R^ cassette fragment as generated with primers Kan^R^-SspI-SD-F and Kan^R^-BsaI-R was fused with a PCR-fragment of part of vector backbone that was generated with primers Kan^R^-AccI-F (5′-ACG TGG CCT GTA GAC GTC CTA AAA G-3′) and Kan^R^-fus-R (5′-GCG AAA CGA TCC TCA TCC TGT AAT ATT ATT GAA GCA TTT ATC AGG G-3′) by fusion PCR. The fused fragment was AccI/BsaI-digested and cloned into the corresponding sites of pUCP20-RHA1, resulting in pUCP20-RHA2. To use *gfp* as a reporter for the rhamnose-inducible system, we cloned the above described SpeI/SbfI-digested *gfp* fragment into the corresponding sited of pUCP20-RHA2, resulting in pUCP20-RHA2-*gfp*.

### Fluorescence microscopy and flow cytometry

For microscopy, 2 μl cell culture was put on an agarose slide. Epifluorescence microscopy was carried out using a Zeiss Axio Imager.M2 (Zeiss, Jena) using 498 nm light for excitation and 516 nm for emission and filter set 13 for GFP fluorescence detection. Photos were taken at × 1000 magnification (Plan-Neofluar × 100 N.A. 1.3 objective) using an AxioCam MRm camera (Zeiss, Jena) and an illumination time of 30 s to obtain comparable results. For flow cytometry, cells were fixed using formaldehyde that was freshly prepared from paraformaldehyde. A total of 1 ml cell culture was harvested at 16,000×*g* for 2 min at room temperature and resuspended in 1 ml PBS containing 2% (w/v) formaldehyde, and incubated for 10 min at room temperature. Next, the cells were sedimented at 16,000×*g* for 2 min at room temperature and resuspended again in 1 ml PBS supplemented with 20 mM Tris-HCl (pH 7.5). The OD600 was measured and adjusted to 0.01; samples were placed on ice and used for analyses by flow cytometry according to the manufacturer protocol (Guava EasyCyte Flow Cytometer, Merck, Darmstadt).

## Results

### The selection and construction of the *antR*/*P*_*antA*_ regulated expression system

In physiological studies in *P. fluorescens* strain A506, we noted the need for a regulated expression system in pseudomonads that permitted the adjustment of distinct lower or higher protein abundancies (Ringel et al. 2016, 2017, 2018). We screened the known genome of this strain for promoters that are likely to be regulated by an AraC family regulator and that are regulating the expression of genes involved in the degradation of small and cheap molecules that could serve as inducers for the regulator. We wanted to avoid the use of sugars as inducers that may interfere with sugar metabolism or require additional uptake systems for non-metabolized sugars. In the *Pseudomonas* genome database (pseudomonas.com), 45 of the genome-encoded proteins of *P. fluorescens* A506 are annotated as AraC family regulators (Suppl. Table S1). Among these, the putative regulator PflA506_4486 is encoded upstream of an operon that is predicted to be responsible for anthranilate degradation. PflA506_4486 has 61% identity to AntR of *P. aeruginosa* PAO1 (Suppl. Fig. S1), which has been demonstrated to be specifically induced by anthranilate, an intermediate of tryptophan degradation found in any bacterium (Choi et al. 2011). We thus decided to use the AntR regulated *P*_*antA*_ promoter for a recombinant expression system in *P. fluorescens* A506, and to compare this with the rhamnose-induced expression system, which is currently preferred for *P. aeruginosa* and *P. putida* (Meisner and Goldberg 2016; Jeske and Altenbuchner 2010).

### The anthranilate-regulated pUCP20-ANT system is advantageous over the rhamnose-regulated system for gene expression in *Pseudomonas fluorescens*

It has never been examined whether an anthranilate-dependent *P*_*antA*_ promoter is suitable to induce a homogeneous gene expression in cell populations and whether it can be used to adjust specific expression levels in *Pseudomonas* cultures. To study this, we cloned the *P*_*antA*_ promoter in pUCP20, a shuttle vector that replicates in *Escherichia coli* as well as in pseudomonads (West et al. 1994). We generated vectors with two different resistance cassettes and with a *gfp* reporter gene or alternatively with a multiple cloning site to facilitate the introduction of desired coding regions. We named this anthranilate-regulated expression system series pUCP20-ANT vectors (Table 1). For comparison, we constructed a rhamnose-inducible *P*_*rhaB*_ promoter–based GFP production system in the same vector (pUCP20-RHA vector). This rhamnose-inducible system originates from *Escherichia coli*. It requires—beside the promoter region—the two regulatory proteins RhaS and RhaR, which are encoded in divergent direction immediately upstream of the *P*_*rhaBAD*_ promoter (Egan and Schleif 1993). The rhamnose-inducible system has been found to function better than the still often used *E. coli P*_*araB*_-based system in *P. aeruginosa* (Meisner and Goldberg 2016), and has since then been successfully used in *P. aeruginosa* (Sonnabend et al. 2020).

**Fig. 2:**
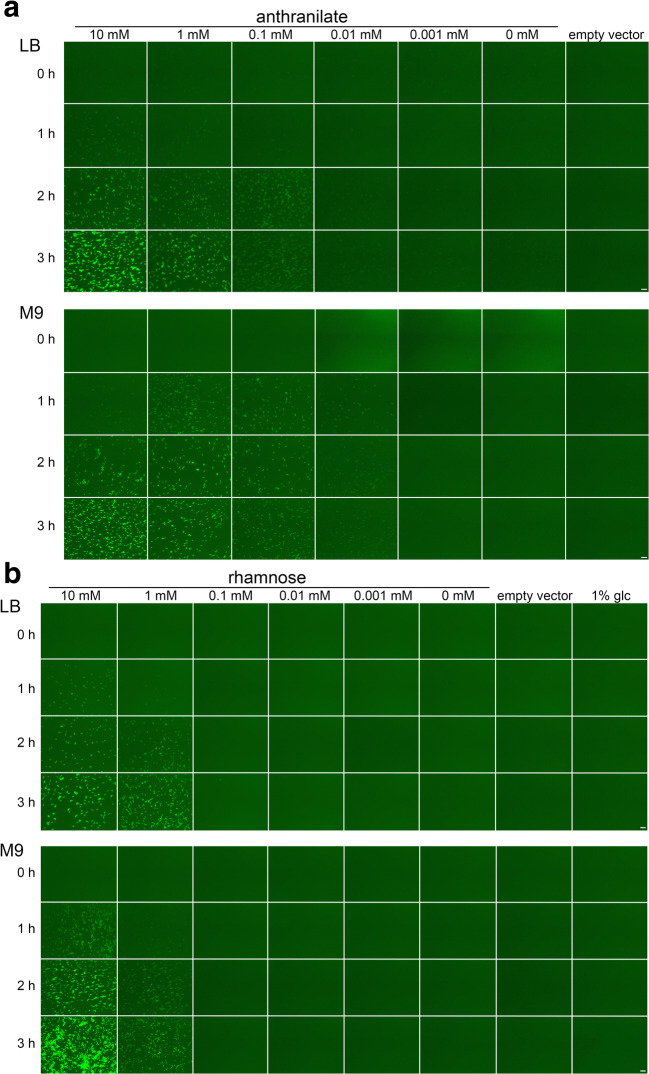
Analysis of GFP reporter fluorescence in anthranilate- or rhamnose-regulated expression systems by epifluorescence microscopy. **a** Analysis of GFP fluorescence in *P. fluorescens* A506 carrying the pUCP20-ANT2-*gfp* expression system. Anthranilate concentrations are indicated at the top, time points at the left of the micrographs. The upper set of micrographs is taken from cultures in LB medium, the lower set from cultures in M9 medium. The empty vector control contained plasmid pUCP20-ANT2. **b** Analysis of GFP fluorescence in *P. fluorescens* A506 carrying the pUCP20-RHA2-*gfp* expression system. Anthranilate concentrations are indicated at the top, time points at the left of the micrographs. The upper set of micrographs is taken from cultures in LB medium, the lower set from cultures in M9 medium. Empty vector control contained plasmid pUCP20-RHA2. For comparison, a glucose-repressed rhamnose system was included in the analyses (1% glc)

At first, we analyzed *gfp* expression in *P. fluorescens* strain A506 by fluorescence microscopy 1 h, 2 h, and 3 h after induction with inducer concentrations ranging from 10 to 0.001 mM (Fig. 2). Cells were grown either in LB or in M9 minimal medium. With 10 mM anthranilate as inducer, fluorescence increased in both media over time to the highest levels at the 3-h time point, and 1 mM and 0.1 mM inducer gave lower fluorescence that was similar at 2-h and 3-h time points, indicating that intermediate expression levels could be achieved that were stable for hours (Fig. 2a). In contrast, rhamnose induced *gfp* gene expression strongly at 10 mM, resulting in an increase of fluorescence over time (Fig. 2b). There was also some expression after 3 h with 1 mM rhamnose detectable. However, there was no clear fluorescence detectable at lower inducer concentrations.

**Fig. 3:**
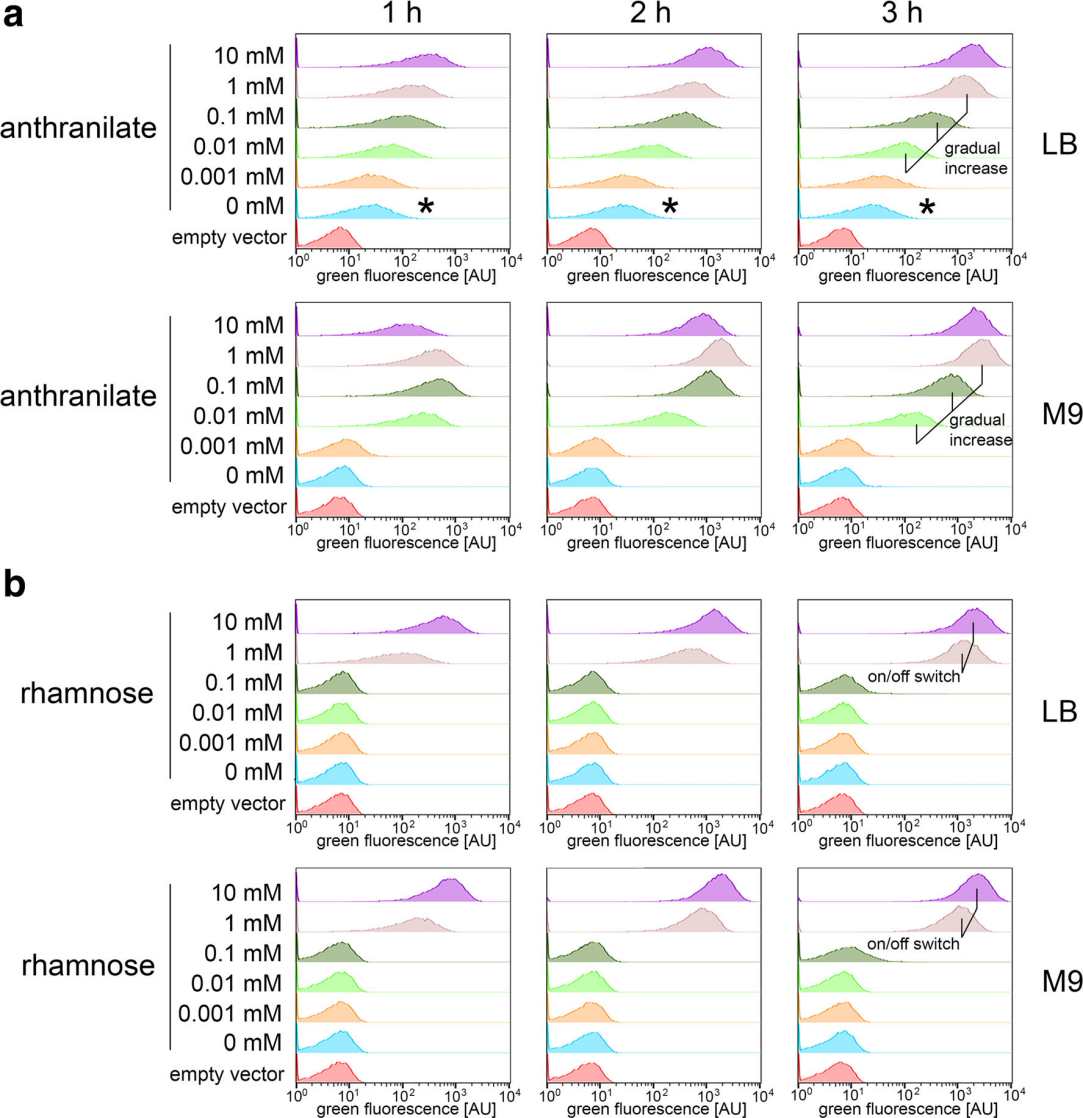
Flow cytometry analyses of anthranilate- and rhamnose-inducible expression systems in *P. fluorescens*. **a** Flow cytometry analysis of GFP reporter fluorescence of strain *P. fluorescens* A506/pUCP20-ANT2-*gfp* growing in LB medium (upper diagrams) or M9 medium (lower diagrams) in response to indicated anthranilate concentrations after 1, 2, and 3 h of induction. As empty vector control, plasmid pUCP20-ANT2 was used. **b** Flow cytometry analysis of GFP reporter fluorescence of strain *P. fluorescens* A506/pUCP20-RHA2-*gfp* growing in KB medium (upper diagrams) or M9 medium (lower diagrams) in response to indicated rhamnose concentrations after 1, 2, and 3 h of induction. As empty vector control, plasmid pUCP20-RHA2 was used. The tuned expression levels are indicated by lines in the 3-h diagram

Although we had strictly used the identical parameters for the recording of the fluorescence micrographs, such data could only give a first hint. We thus carried out flow cytometry to compare the distribution of cellular fluorescence in the population (Fig. 3). The detection of fluorescence was by far more sensitive by flow cytometry.

When grown in LB medium, the *P*_*antA*_ promoter system reached very high fluorescence levels in the whole population with 1 mM anthranilate, and with 0.1 mM and 0.01 mM anthranilate, we observed stable intermediate expression levels (Fig. 3a). With 0.001 mM inducer, we found the same fluorescence as in a control without inducer, indicating that the useful range of anthranilate inducer concentration is between 1 and 0.01 mM in LB medium. The fluorescence in LB medium showed a very low but clearly detectable leakiness, being without inducer about twofold higher than in the empty vector control after 3-h induction. When grown in M9 minimal medium, this little leakiness was not detectable anymore, and the useful range of inductor concentrations was again between 1 and 0.01 mM anthranilate, with 1 mM for full induction and 0.01 mM for lowest induction. At 0.01 mM anthranilate, there was no induction, as in the case of no inducer or the empty vector control. Together, these data showed that the anthranilate-inducible system permits a fine tuning of expression levels in *P. fluorescens*, and M9 minimal medium can be used to achieve non-detectable leakiness with the GFP reporter under aerobic growth on glucose.

With the rhamnose system, the induction behavior was very different. When grown in LB medium, the induction of the *P*_*rhaB*_ promoter system was strong with 10 mM rhamnose as well as with 1 mM rhamnose after 3 h of induction, whereas there was no induction with 0.1 mM rhamnose or lower concentrations (Fig. 3b). Moreover, the induction by 10 mM or 1 mM rhamnose was continuously increasing over time to reach full or nearly full induction, the highest level being reached only faster with 10 mM than with 1 mM, but there was no intermediate fluorescence level stably established. The same switch occurred with this *P*_*rhaB*_ promoter system in M9 minimal medium, indicating that the absence of complex constituents did not have any influence. Together, these data showed that, in *P. fluorescens*, only at higher rhamnose levels can this inducer switch the promoter on, and this switch occurs in the whole cell population.

Some larger peak width at lower inducer concentrations (also in case of leakage) or prior to steady-state time points (with anthranilate as well as rhamnose systems) is likely due to stochastically initiated expressions at low intracellular inducer concentrations when cells can switch between “on” and “off” states without forming distinct populations. Such low intracellular inducer concentrations exist either for hours (with low externally added anthranilate or with intrinsically formed anthranilate) or at the onset of the induction (with high inducer concentrations). Due to the logarithmic scale in these plots, these broader peaks reflect only slight absolute variations.

**Fig. 4:**
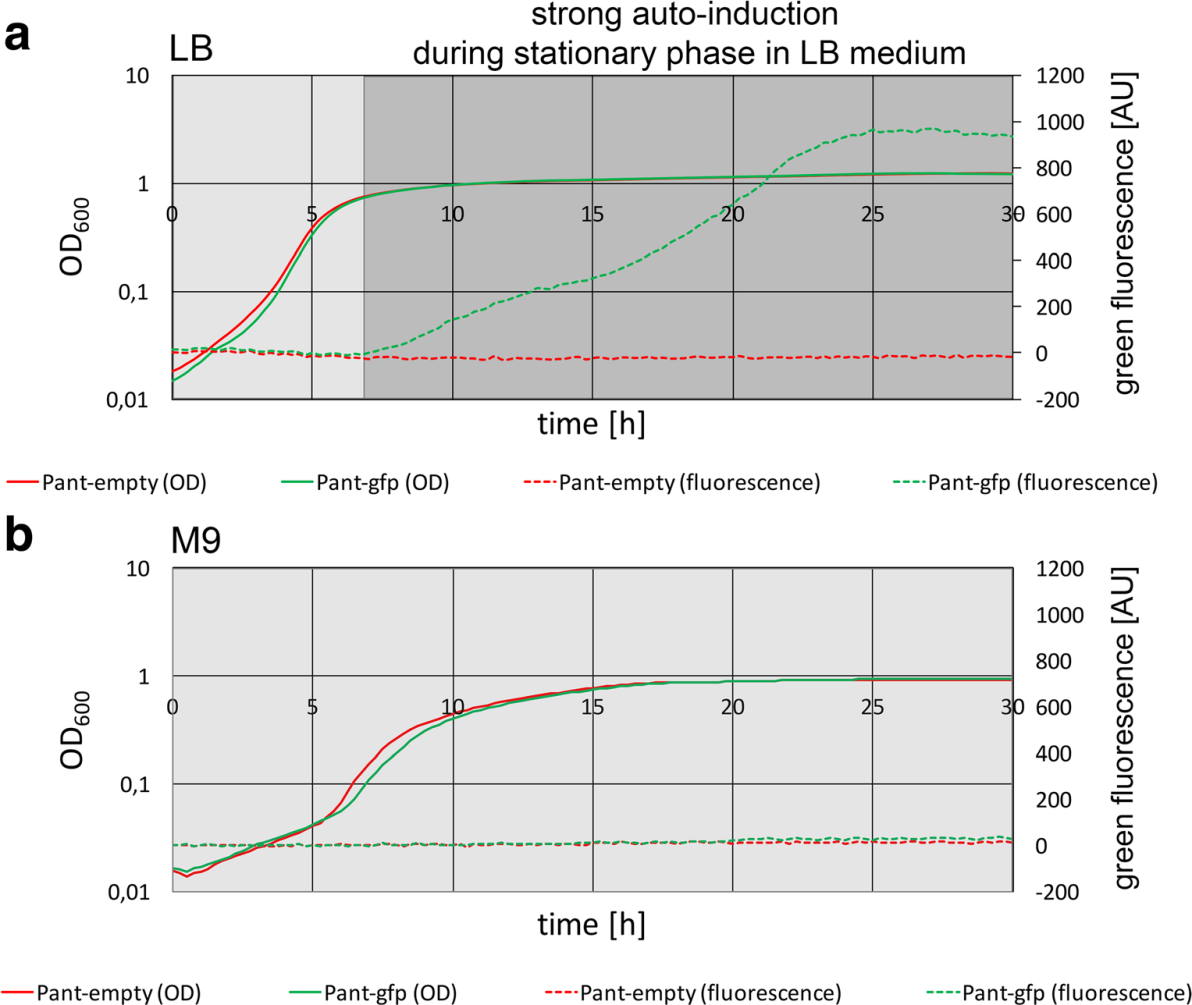
Analysis of autoinduction during growth of *P. fluorescens*. Reporter GFP fluorescence development of strain *P. fluorescens* A506/pUCP20-ANT2-*gfp* during growth on LB (**a**) or M9 (**b**) medium without externally added inducer. Plotted are, as indicated, OD600 and green fluorescence, with excitation at 488 nm and detection at 509 nm

The observation of some basal promoter activity in LB medium without added inducer prompted us to examine the potential development of inherent promoter activity with the anthranilate-inducible system during growth in LB medium or M9 minimal medium (Fig. 4). Autonomous expression from the *P*_*antA*_ promoter system was extremely low during exponential growth and strongly induced at the beginning of stationary growth in LB medium (Fig. 4a). This induction during stationary growth was biphasic. Notably, there was no inherent induction of the *P*_*antA*_ promoter when cells were grown in M9 medium (Fig. 4b), which agrees with the above data (Fig. 3).

### The *antR*/*P*_*antA*_ regulated system can also be used in *Pseudomonas aeruginosa* and in *Pseudomonas putida*

**Fig. 5:**
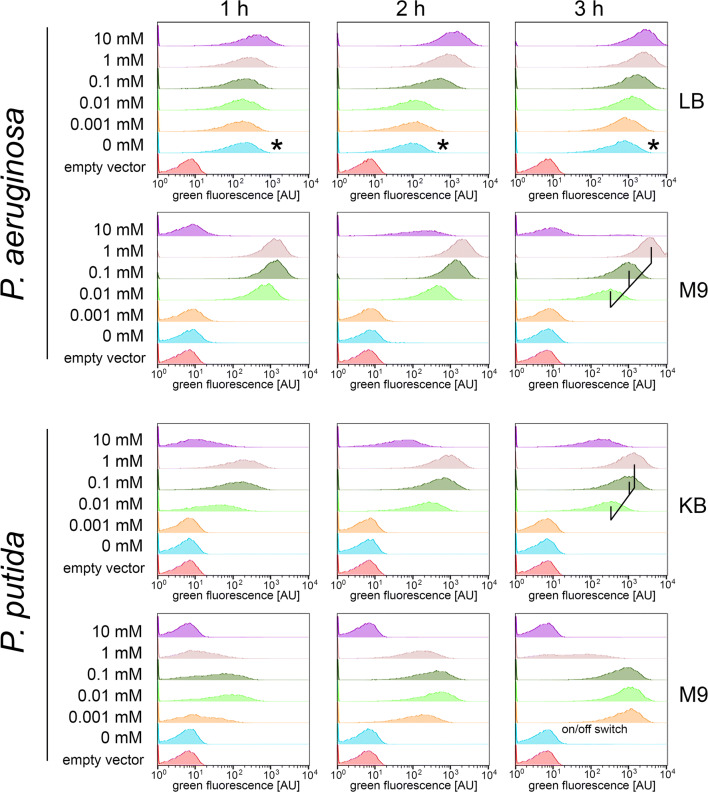
Flow cytometry analyses of anthranilate-inducible expression systems in *P. aeruginosa* and *P. putida*. **a** Flow cytometry analysis of GFP reporter fluorescence of strain *P. aeruginosa* PAO1/pUCP20-ANT1-*gfp* growing in LB or M9 medium in response to indicated anthranilate concentrations after 1, 2, and 3 h of induction. As empty vector control, plasmid pUCP20-ANT1 was used. Asterisks indicate autoinduction. **b** Flow cytometry analysis of GFP reporter fluorescence of strain *P. putida* DSM291^T^/pUCP20-ANT2-*gfp* growing in KB or M9 medium in response to indicated anthranilate concentrations after 1, 2, and 3 h of induction. As empty vector control, plasmid pUCP20-ANT2 was used. The tuned expression levels are indicated by lines in the 3-h diagram

As the *P*_*antA*_ promoter–dependent expression system turned out to be useful for *P. fluorescens*, we wanted to know whether this system behaved similarly in other pseudomonads. We chose the commonly used reference strain *P. aeruginosa* PAO1 and *P. putida*–type strain DSM291^T^ for these comparative analyses. Like in the case of *P. fluorescens*, we tested the expression system with the strains grown in either a complex medium or a minimal medium (Fig. 5). *P. aeruginosa* was grown in LB and M9 medium, whereas *P. putida* was grown in King’s B and M9 medium; anthranilate was used at concentrations ranging from 0.001 to 10 mM for induction, and this was compared with controls without inducer or the empty vector control (lacking the *gfp* gene).

When grown in LB medium, *P. aeruginosa* showed a strong autoinduction, independent from the presence of externally added inducer, indicating that *P. aeruginosa* generates anthranilate in this complex medium (Fig. 5a). In contrast, there was no such autoinduction observed with M9 minimal medium, and anthranilate could induce expression when added at a concentration of 0.01 mM or higher. Fluorescence with 0.01 mM anthranilate had its maximum at the 1-h time point and then slowly decreased over time, suggesting a detectable influence of turnover of anthranilate on induction. Induction with 0.1 mM anthranilate resulted in higher fluorescence that was more stable over time, although there was again some reduction of fluorescence at the 3-h time point. With 1 mM anthranilate, induction was highest and in this case fluorescence even somewhat increased over time. Anthranilate of 10 mM had clear toxic effects in M9 medium, as only low fluorescence was detectable at the 2-h time point which had disappeared already at the 3-h time point. Most likely, high concentrations of anthranilate can act as uncoupler negatively on the energetization of the cells. Therefore, anthranilic acid–dependent induction is useful in *P. aeruginosa* M9 minimal medium at inducer concentrations between 0.01 and 1 mM, which permits some fine tuning of the expression level.

For *P. putida*, King’s B medium gave stable expression levels, whereas expression in M9 minimal medium shifted over time (Fig. 5b). In King’s B medium, there was no induction with 0.001 mM, low induction with 0.01 mM, and higher induction with 0.1 mM anthranilate. There was no further boost of induction at 1 mM anthranilate, and 10 mM inducer caused toxic effects. In M9 medium, inductions were observed already with 0.001 mM anthranilate, but there was hardly any difference between 0.001 and 0.1 mM inducer, shifting always to strong induction after 3 h. Toxic effects were already obvious at 1 mM anthranilate, and cells did not even grow anymore at 10 mM anthranilate.

## Discussion

Our data indicate that the anthranilate-inducible *P*_*antA*_ promoter of *P. fluorescens* A506 can be useful for regulated gene expression in the three tested *Pseudomonas* species *P. fluorescens*, *P. aeruginosa*, and *P. putida*. Distinct media had distinct effects on autoinduction background and on anthranilate toxicity in the tested strains. LB medium resulted in autoinduction in both tested cases (*P. fluorescens* and *P. aeruginosa*), and LB therefore seems not to be recommendable for the system. The reason for the autoinduction is likely the degradation of the tryptophan-containing peptides in LB as energy source in that medium, which results in intrinsic anthranilate production during exponential and stationary growth when tryptophan is degraded. M9 medium turned out to be suitable for these two species, as there was hardly any detectable autoinduction in this peptide-free medium, and stable induction levels could be achieved at several inducer concentrations. However, *P. putida* was more sensitive towards anthranilate than the other species, and in M9 medium, this sensitivity was most momentous, with cells not growing at all in the presence of 10 mM anthranilate. Importantly, for *P. putida*, often used King’s B medium was suitable for anthranilate-induced expression, as this medium did not result in autoinduction, just as did M9 minimal medium, and a range of distinct expression levels could be achieved. In the case of *P. aeruginosa*, it is important to consider a potential influence of anthranilate addition on the formation of PQS in physiological analyses that are carried out under iron limitation (Farrow and Pesci 2007). Under these specific conditions, which are typical for host environments, anthranilate can be converted by *pqsABCDE* and *pqsH* gene products to this acylated quinolone that is involved in multiple aspects of virulence, including iron acquisition, cytotoxicity, and quorum sensing (Lin et al. 2018). Only *P. aeruginosa* possesses *pqs* genes and produces PQS (pseudomonas.com, Winsor et al. 2016). A related aspect is that the small RNAs PrrF1 and PrrF2, which regulated many genes in response to iron deficiency, inhibit translation of the *antR* mRNA in *P. aeruginosa*. This serves to suppress anthranilate degradation, thereby providing the biosynthetic precursor for PQS (Djapgne et al. 2018). PrrF1/2 effects are modulated by Hfq and Crc in conjunction with the target-titrating sRNA CrcZ, which coordinates iron acquisition with carbon availability (Sonnleitner et al. 2017; Sánchez-Hevia et al. 2018). While the PrrF1/F2/Hfq/Crc/CrcZ regulatory system exists in many pseudomonads, the specific inhibition of *antR* mRNA translation is restricted to *P. aeruginosa* as the others do not synthesize PQS. Accordingly, the PrrF1/2 binding site is not conserved in the 5′-UTR of *P. fluorescens antR* mRNA, and the pUCP20-ANT expression system will therefore be most likely unaffected by these sRNAs.

In conclusion, if the system is to be used with new isolates, we can only recommend to test different media to clarify the aspects of anthranilate sensitivity and autoinduction. As we could establish useful conditions for tightly regulated anthranilate-induced expression in any of the three species, it is likely that the system will be useful also in other strains and species. It has to be kept in mind that a leakage below our detection level may well be sufficient and desired for complementation of enzyme functions, as often few enzyme molecules per cell can provide sufficient turnover for metabolic pathways. As in any expression system, altered experimental setups need to be considered to influence the system, such as limited oxygen supply, altered nutrients, or growth in biofilms.

Anthranilate is a ubiquitous metabolic intermediate of tryptophan degradation, and it apparently can readily enter cells and therefore most likely can be used in any species studied. As in the case of the structurally related benzoic acid, the growth inhibitory effect of high anthranilate concentrations could be due to an acidification of the cytoplasm (Salmond et al. 1984), but this will not be caused by the carboxyl group (pKa ~ 2.2) but rather by the neighboring amino group (pKa ~ 4.9) that can enter the cytoplasm in a protonated form from the more acidic periplasmic side of the membrane and release protons inside. To our knowledge, there is no specific anthranilate transporter known. As anthranilate is charged at any physiological pH, it may pass the membrane bilayer via low-specific small acid or amino acid transporters. It has been found that anthranilate derivatives efficiently inhibit sodium/dicarboxylate transporters, which suggests that non-modified anthranilate might be transported by such transporters (Pajor and Randolph 2007). However, anthranilate is secreted in large quantities by rhizobia under iron limitation and can be used for iron uptake (without being assimilated under iron-limiting conditions), indicating that there likely exist anthranilate-specific secretion and uptake pathways, at least in these species (Rioux et al. 1986).

The anthranilate-based expression system can give almost constant lower expression levels at lower inducer concentrations (Figs. 3 and 5), which can be useful for physiological assays in liquid cultures. With the rhamnose-inducible system, we could not achieve such a constant expression. Instead, at any rhamnose concentration that could induce the system, the expression level increased over time to a similar maximum level at 3 h. Rhamnose therefore appears not to be suitable for tuning the expression level in *P. fluorescens* A506. In *P. putida*, the rhamnose-regulated system has been tested with high rhamnose concentrations (~ 12 mM) and thereby reached high expression levels (Jeske and Altenbuchner 2010). Also for *P. aeruginosa*, the heterologous *E. coli* rhamnose system has been reported to function well (Meisner and Goldberg 2016). Neither in *P. putida* nor in *P. aeruginosa* where it has been addressed whether the cultures are homogeneously induced, especially at intermediate expression levels. Nevertheless, when just high expression levels are required, the rhamnose-inducible system and the anthranilate-inducible system are both good options, and it can be important to have anthranilate an alternative to sugar.

We found that fluorescence intensity as achieved with anthranilate- or rhamnose-inducible systems was always distributed in a single Gaussian distribution, not showing two populations of cells such as found in all-or-nothing expression systems that have the transporter for the inducer under control of the inducer itself, such as that found in the case of the arabinose system in *E. coli* (Siegele and Hu 1997; Khlebnikov et al. 2000). For the rhamnose system, this was expected as *P. fluorescens* has no annotated rhamnose-specific uptake system, nor does it encode homologs of the rhamnose operon genes of *E. coli*. The requirement of high rhamnose concentrations for the induction of the heterologous rhamnose system in *P. fluorescens* A506 may thus be explained by the absence of a rhamnose-specific uptake system and therefore the employment of other transporters with only low affinity for rhamnose. Certainly such transporters are not under control of rhamnose as inducer. In the case of the anthranilate system, the data suggest that the natural metabolite anthranilate can be taken up by most if not all strains also at lower concentrations, which is a clear advantage of anthranilate over rhamnose. In conclusion, the herein described anthranilate-based expression system has important advantages over other systems: It permits low as well as high gene expression levels; it is applicable for a wide range of pseudomonads; growth conditions can be found under which this system shows no detectable leakage; and the inducer anthranilate is a cheap, non-toxic, and stable compound. We hope that future physiological and biotechnological applications in many pseudomonads will benefit from this system.

## Supplementary Information

ESM 1 (PDF 595 kb)

## Data Availability

All vectors generated in this study can be obtained from the corresponding author.
