# Supplementary material for: A tunable anthranilate-inducible gene expression system for Pseudomonas species
Source: Appl Microbiol Biotechnol. 2020 Dec 3;105(1):247–58. doi: 10.1007/s00253-020-11034-8 (PMC7778614; doi:10.1007/s00253-020-11034-8)
Supplement: Supplementary file 1 — (PDF 595 kb) [file 253_2020_11034_MOESM1_ESM.pdf]

Supplementary Material

Journal name:

Applied Microbiology and Biotechnology

Manuscript title:

A tunable anthranilate-inducible gene expression system for *Pseudomonas* species

Authors names:

Lena Hoffmann, Michael-Frederick Sugue, and Thomas Brüser*

Authors affiliation and address:

Institute of Microbiology, Leibniz University Hannover, Herrenhäuser Str. 2, 30419 Hannover, Germany

Corresponding author:

Thomas Brüser
E-Mail: brueser@ifmb.uni-hannover.de
Phone: +49 (0)511 7625945
Fax: +49 (0)511 7625287

Supplement Table S1

List of the 45 AraC-family proteins of *Pseudomonas fluorescens* A506, as annotated at “pseudomonas.com” (Winsor et al. 2016). The AntR homolog PflA506_4486 is highlighted.

locus tag	orf length (nt)	protein size (residues)	annotation
PflA506_0206	1005	334	AraC family transcriptional regulator
PflA506_0557	789	262	AraC family transcriptional regulator
PflA506_0688	810	269	AraC family transcriptional regulator
PflA506_0827	996	331	AraC family transcriptional regulator
PflA506_1989	969	322	AraC family transcriptional regulator
PflA506_2163	969	322	AraC family transcriptional regulator
PflA506_2234	1176	391	AraC family transcriptional regulator
PflA506_2273	939	312	AraC family transcriptional regulator
PflA506_2310	771	256	AraC family transcriptional regulator
PflA506_2318	897	298	AraC family transcriptional regulator
PflA506_2342	1038	345	AraC family transcriptional regulator
PflA506_2443	894	297	AraC family transcriptional regulator
PflA506_2585	1017	338	AraC family transcriptional regulator
PflA506_2600	729	242	transcriptional regulator, AraC family
PflA506_2609	810	269	AraC family transcriptional regulator
PflA506_2816	810	269	AraC family transcriptional regulator
PflA506_2822	1011	336	AraC family transcriptional regulator
PflA506_2841	906	301	AraC family transcriptional regulator
PflA506_2889	924	307	AraC family transcriptional regulator
PflA506_2892	822	273	AraC family transcriptional regulator
PflA506_2972	840	279	AraC family transcriptional regulator
PflA506_3058	846	281	AraC family transcriptional regulator
PflA506_3074	399	132	AraC family transcriptional regulator
PflA506_3077	951	316	AraC family transcriptional regulator
PflA506_3080	972	323	AraC family transcriptional regulator
PflA506_3108	948	315	AraC family transcriptional regulator
PflA506_3113	777	258	AraC family transcriptional regulator
PflA506_3120	996	331	AraC family transcriptional regulator
PflA506_3125	789	262	AraC family transcriptional regulator
PflA506_3292	1035	344	AraC family transcriptional regulator
PflA506_3408	942	313	AraC family transcriptional regulator
PflA506_3557	1086	361	DJ-1/PfpI family protein / transcriptional regulator, AraC family
PflA506_3578	996	331	AraC family transcriptional regulator
PflA506_3768	750	249	AraC family transcriptional regulator
PflA506_3984	894	297	AraC family transcriptional regulator
PflA506_4025	999	332	AraC family transcriptional regulator
PflA506_4167	1059	352	AraC family transcriptional regulator
PflA506_4211	741	246	AraC family transcriptional regulator
PflA506_4269	930	309	AraC family transcriptional regulator
PflA506_4438	876	291	AraC family transcriptional regulator
**PflA506_4486**	**993**	**330**	**AraC family transcriptional regulator**
PflA506_4789	972	323	AraC family transcriptional regulator
PflA506_4968	945	314	AraC family transcriptional regulator
PflA506_5132	966	321	AraC family transcriptional regulator
PflA506_5358	993	330	AraC family transcriptional regulator

Supplement Figure S1

Clustal Omega (Madeira et al. 2019) sequence alignment of PflA506_4486 from *P. fluorescens* A506 with AntR from *P. aeruginosa* PAO1.

```
PflA506_4486      -----MSSQTRDIHIQRFDLEGARSWMSGICGPHRLATATPERLRFHHSANVFKSRATTL 55
PAO1_AntR         MMRTHPVADRGDLHADHLDLAAARSWMSKVCGPHRLEAASPGLVQFQHHGNVLKSMCTTL 60
                         ::  *:* :::** .****** :****** :*:*  ::*:* .**:** .***

PflA506_4486      GVIEYGTDVTIDIEDAEHFRSYSLSLPLVGEQELSKNGERLSSNRDQGVIISPNEHQVLA 115
PAO1_AntR         GYIGYGTDVTITVEDAAAFNAYSLSLPLSGEQELCRGGLRLLSDVRRGVIIAPNERQELS 120
                  * * ******* :***  *.:******* *****.:.* ** *:  :****:***:* *:

PflA506_4486      ISGDCRKLQVVITRAAMSESLEGLLQRPIDAPLRFESVMDAVDGAPASWWRMARYFIAEL 175
PAO1_AntR         IAGDCRKLQVVIGRTAMRKVLEEMLQRPIDTPLRFDPEMDALDGASASWWRTVRHISEE- 179
                  *:********** *:** : ** :******:****:  ***:*** ***** .*::  *

PflA506_4486      ECSSELYEQAAFTRDLESSLIKGLILAQPNNYSEELREVLGVKLPHYLIRARQFIHDNAR 235
PAO1_AntR         MARSELYAQAFFSSDLERALIKGLILAQPNNYSEALQQGLGGRPPHYLLRAREFLQANAR 239
                   . **** ** *: *** :*************** *:: ** : ****:***:*:: ***

PflA506_4486      EVLHLEDLEAAAGVSRFKLFDAFRKYFALSPMAYLKKHRLGAVRQEILEQGSMRTISEIA 295
PAO1_AntR         ETLSLEDVERAAGVSRFKLFEGFRRYFGVSPMSYLKHYRLAAVREEILASGGARSISTIA 299
                  *.* ***:* **********:.**:**.:***:***::**.***:*** .*. *:** **

PflA506_4486      LGWGFTHLGRFSAEYRKLFDESPSQTLQRKRLRIT 330
PAO1_AntR         LGWGFSHLGRFSVDYRKRFEETPSMTQRRAARRS- 333
                  *****:******.:*** *:*:** * :*   *
```

Supplement References

Madeira F, Park YM, Lee J, Buso N, Gur T, Madhusoodanan N, Basutkar P, Tivey ARN, Potter SC, Finn RD, Lopez R (2019) The EMBL-EBI search and sequence analysis tools APIs in 2019. Nucleic Acids Res 47:W636-W641

Winsor GL, Griffiths EJ, Lo R, Dhillon BK, Shay JA, Brinkman FSL (2016) Enhanced annotations and features for comparing thousands of *Pseudomonas* genomes in the Pseudomonas genome database. Nucleic Acids Res 44:D646-53
